# Prevalence and factors associated with reported adverse-events among patients on multi-drug-resistant tuberculosis treatment in two referral hospitals in Uganda

**DOI:** 10.1186/s12879-023-08085-3

**Published:** 2023-03-10

**Authors:** Paul Mukama Ategyeka, Michael Muhoozi, Racheal Naturinda, Peter Kageni, Carol Namugenyi, Amos Kasolo, Stevens Kisaka, Noah Kiwanuka

**Affiliations:** 1 College of Health Sciences School of Public Health, Makerere University, Kampala, Uganda; 2 Makerere University Center for Health and Population Research, Kampala, Uganda; 3 Mulago National Referral Hospital TB ward 5 and 6, Kampala, Uganda; 4 Mbarara Regional Referral Hospital TB ward, Mbarara, Uganda; 5 College of Health Sciences Department of Pharmacy, Makerere University, Kampala, Uganda; 6 College of Veterinary Medicine, Animal Resources and Biosecurity, Makerere University, Kampala, Uganda; 7 Center for Epidemiological Modelling and Analysis (CEMA), University of Nairobi Institute of Tropical and Infectious Diseases, Nairobi, Kenya

**Keywords:** Multi-drug resistant, Tuberculosis, Adverse-events

## Abstract

**Background:**

Multi-drug-resistant tuberculosis (MDR-TB) treatment involves toxic drugs that cause adverse events (AEs), which are life-threatening and may lead to death if not well managed. In Uganda, the prevalence of MDR-TB is increasingly high, and about 95% of the patients are on treatment. However, little is known about the prevalence of AEs among patients on MDR-TB medicines. We therefore estimated the prevalence of reported adverse events (AEs) of MDR-TB drugs and factors associated with AEs in two health facilities in Uganda.

**Methods:**

A retrospective cohort study of MDR-TB was conducted among patients enrolled at Mulago National Referral and Mbarara Regional Referral hospitals in Uganda. Medical records of MDR-TB patients enrolled between January 2015 and December 2020 were reviewed. Data on AEs, which were defined as irritative reactions to MDR-TB drugs, were extracted and analyzed. To describe reported AEs, descriptive statistics were computed. A modified Poisson regression analysis was used to determine factors associated with reported AEs.

**Results:**

Overall, 369 (43.1%) of 856 patients had AEs, and 145 (17%) of 856 had more than one. Joint pain (244/369, or 66%), hearing loss (75/369, or 20%), and vomiting (58/369, or 16%) were the most frequently reported effects. Patients started on the 24-month regimen (adj. PR = 1.4, 95%; 1.07, 1.76) and individualized regimens (adj. PR = 1.5, 95%; 1.11, 1.93) were more likely to suffer from AEs. Lack of transport for clinical monitoring (adj. PR = 1.9, 95%; 1.21, 3.11); alcohol consumption (adj. PR = 1.2, 95%; 1.05, 1.43); and receipt of directly observed therapy from peripheral health facilities (adj. PR = 1.6, 95%; 1.10, 2.41) were significantly associated with experiencing AEs. However, patients who received food supplies (adj. PR = 0.61, 95%; 0.51, 0.71) were less likely to suffer from AEs.

**Conclusion:**

The frequency of adverse events reported by MDR-TB patients is considerably high, with joint pain being the most common. Interventions such as the provision of food supplies, transportation, and consistent counseling on alcohol consumption to patients at initiation treatment facilities may contribute to a reduction in the rate of occurrence of AEs.

## Background

Globally, an estimated 4.1% and 19% of new and retreatment tuberculosis (TB) cases respectively [1] are believed to have rifampicin resistance and started on second line anti-TB treatment [2, 3]. The treatment lasts for 18 to 24 months: 6 months of injectables, and the other months the patients receive oral medications [4]. However, they are newer oral regimens. The treatment involves toxic drugs that cause adverse-events (AEs), which can be life threatening and may lead to death if not properly managed [1]. According to studies, the prevalence of adverse events associated with second-line anti-TB drugs ranges from 30 to 90% [5–7]. A study conducted in Ethiopia found that 89.9% of the multi-drug resistant tuberculosis (MDR-TB) patients on treatment had AEs [8].

In 2019, an estimated 88,000 people fell ill with TB in Uganda, and an estimated 15,600 people died [9]. Furthermore, in 2019, of the estimated 1,500 drug-resistant TB (DR-TB) cases, only 559 were diagnosed and started on treatment, and 96% of the patients who started treatment in 2017 completed treatment [10]. It was estimated that 57% of the patients that were started on second-line anti-TB treatment during 2016 experienced AEs [4] which may have contributed to lower treatment success rates, prolonged periods of morbidity, and higher mortality. The MDR-TB guidelines recommend that monthly clinical check-ups be conducted for all patients started on treatment to monitor AEs [4].

MDR-TB patients are started on second-line anti-TB treatment that lasts for 18 to 24 months or 9 to 12 months. The choice of which regimen the patient receives depends on the physicians and pharmacists. The treatment regimen may include: 18–24 months; 6 months of kanamycin (km), levofloxacin (lfx), ethionamide (eto), cycloserine (cs); 18 months of levofloxacin (lfx), ethionamide (eto), cycloserine (cs), or the short-term regimen will last for 9–12 months; 4–6 months of kanamycin (km)- moxifloxacin (Mfx)- prothionamide (Pto)- clofazimine (Cfz)- pyrazinamide (Z)- high-dose isoniazid (Hhigh-dose)- ethambutol (E) /5 months of moxifloxacin (Mfx)- clofazimine (Cfz)- pyrazinamide (Z)-ethambutol (E). [4, 11, 12]. These drugs are taken for longer periods, they are also highly toxic and cause adverse events if patients are not monitored and well managed [13].

Adverse events can be managed through constant monitoring of the patient and minimal modification of the treatment regimen [14]. Studies have shown that kanamycin is the most frequently substituted drug in the regimen [15]. Guidelines recommend that MDR-TB patients receive MDR-TB treatment under the directly observed therapy (DOT) [4]. During this time, patients are admitted at start of treatment for a period of 2 weeks to a month, or until the culture converts, and then they are discharged to the community, where they continue to receive treatment under DOT from a lower-level facility near their home of choice.

2% of MDR-TB patients stopped treatment, and 30% required removal of the suspected drugs from the regimen due to AEs because they were monitored daily while taking their treatment [16]. If the patients are mismanaged, it can easily cost them their lives or result in permanent disabilities like hearing loss. It is important to understand and know the number of people affected by AEs of second-line anti – TB treatment. However, there is relatively limited research about prevalence and incidence of AEs related to MDR – TB treatment, and most of the studies have focused on the factors associated with the AEs. As a result, the purpose of this study was to determine and describe the prevalence of reported AEs of second-line drugs, as well as the factors associated with them.

## Methods

### Study design

The study was a retrospective cohort study that employed quantitative research methods among MDR-TB patients receiving or who received second line anti-TB drugs. This involved reviewing the records of MDR-TB patients enrolled on second line TB drugs. Such records included the drug-resistant management information system (DR-TB MIS) that has most of the patient information, and the patients’ files which are kept at the initiation facilities in locked cabinets.

### Study setting

The study was conducted in Mulago national referral hospital (NRH) (TB ward, that is, wards 5 and 6) and Mbarara regional referral hospital (RRH) (TB ward) in Uganda. Mulago NRH is the largest public hospital in Uganda. It’s located on Mulago Hill in the northern part of Kampala, less than five kilometers (km) from Kampala’s central business district. The MDR-TB site in Mulago NRH serves the central region that includes districts such as Mpigi, Luwero, Kayunga, Buikwe, Kampala divisions, and Wakiso. It leads the national MDR-TB panel and has the greatest number of patients initiated on treatment. Mbarara RRH is located in Mbarara district, Ankole sub-region within the central business district by road, it is approximately 268 km south-west of Kampala, Uganda’s capital city. The hospital has an MDR-TB site that serves districts such as Mbarara, Isingiro, Bushenyi, Kiruhura, Ibanda, Ntugamo, Sheema, and Mitooma. The hospital serves a population of over four million people and has a bed capacity of over 350 beds. Both facilities are responsible for coordinating and training follow up facilities (FUFs) in administering DOTs to MDR-TB patients in their respective regions. Both Mulago NRH and Mbarara RRH were selected because of the great numbers of MDR-TB patients that are seen at these two facilities. The facilities had updated records of their MDR-TB patients compared to other MDR-TB facilities.

### Study Population

The study population comprised all confirmed MDR-TB patients who were started on second-line anti-TB treatment during the period of 1st Jan, 2016 and 31st Dec, 2020. These patients included those who were documented as having started, completed, or were still on treatment. This period was chosen because the MDR-TB program had been fully initiated in these two facilities. The study excluded all patients that were transferred to Mulago NRH-TB and Mbarara RRH. This was because the review of these patients’ records is done at their former initiation site which may have caused double counting.

### Data collection Procedure

Data was downloaded into Microsoft excel from the MDR-TB MIS on the district health information system 2 (DHIS 2) platform. In case of any missing data, data was extracted from the patients’ files so that it would fill in the missing gaps in the data in order to have a complete data set in MS-excel. The data was then exported to Stata version 14, where data cleaning was done. This was done by identifying the duplicates and transfer ins from other sites which were dropped from the data set. The data categorization and classification of reported AEs were done according to the categorize and classifications within the DR-TB MIS that is, regimen, TB registration group, place of DOT adherence to treatment, and also the categorizes of AEs.

### Data analysis

Data were analyzed using Stata v.14 software. Data on demographic and clinical factors were summarized using frequencies and percentages Additionally, the prevalence of reported AEs among MDR-TB patients was reported as a percentage. To determine the factors associated with reported AEs of taking second line anti-TB drugs, a modified poison generalized linear model (log link with robust standard errors) was used. The model included variables with p < 0.25 at bivariate analysis or variables found to be potentially or known to be associated with the outcome from the literature. Both the unadjusted and adjusted prevalence ratios and corresponding 95% confidence intervals are presented.

Multi-collinearity was done, and no variables were removed due to multi-collinearity, since they had a p-value less than 0.40. Then these variables were selected, their prevalence ratios were adjusted for multivariable analysis. Forward and backward elimination was used to select the variables after considering the p-value of less than 0.05 to come up with a perfect model. Furthermore, additional analysis was done by stratifying by type of patients that is to say by new and retreatment MDR-TB cases (see supplementary file). We were unable to compute for the statistical differences between severity of AEs and other factors because one patient could have more than one AE and they could have different severities, making it difficult to compute.

## Results

### Descriptive statistics

A total of 856 MDR-TB patients’ data were extracted, including 369 (43.1%) patients with AEs. Most MDR-TB patients were male 543 (63.4%), with most of the patients aged 25–34 291 (34%) where the mean age was 34 years (SD of 12.4 years), majority of the patients had a weight of 46-55.9 kg (kg) 320 (37.4%) with a mean weight of 48.3 kg (SD of 25.12 kg). The majority of the patients (354; 41.4%) were single, with 633 (73.9%) working in informal employment and 589 (92.9%) married. The details are shown in Table 1 below.

Most of the MDR-TB patients were new cases 454 (53%) and mostly co-infected with HIV 489 (57.1%). The majority of the patients (457/53.4%) were started on a long-term regimen (LTR), and the majority of them (744/86.1%) received their treatment from follow-up facilities (FUFs). The majority of them 505 (59%) had attended their clinical visits, 690 (80.6%) had received food supplies, and 767 (89.6%) had received transportation. The majority of them 565 (66%) stayed on treatment, and only 287 (33.5%) drank alcohol while on treatment. As seen in Table 1.

**Table 1:** Social –demographic factors for the MDR-TB patients in Mulago NRH and Mbarara RRH

Characteristics	Frequency (Percentage)
	(n = 856)
**Age**	
0–14	39 (4.6%)
15–24	133 (15.5%)
25–34	291 (34%)
35–44	230 (26.9%)
45–54	114 (13.3%)
55+	49 (5.7%)
**Weight**	
1-29.9 kg	77 (9%)
30-35.9 kg	27 (3.1%)
36-45.9 kg	189 (22.0%)
46-55.9 kg	320 (37.4%)
56-69.9 kg	201 (23.5%)
70 kg+	42 (4.9%)
**Sex**	
Male	543 (63.4%)
Female	313 (36.6%)
**Marital Status**	
Single	354 (41.4%)
Married	350 (40.9%)
Separated	152 (17.8%)
**Occupation**	
Working	633 (73.9%)
Not working	223 (26.1%)
**Form of employment**	
Formal	45 (7.1%)
Informal	589 (92.9%)
**Health facilities**	
Mulago NRH	684 (79.9%)
Mbarara RRH	172 (20.1%)
**Year of treatment**	
2015	114 (13%)
2016	166 (19%)
2017	136 (16%)
2018	166 (19%)
2019	182 (21%)
2020	92 (11%)
**Co-Infected with HIV**	
No	367 (42.9%)
Yes	489 (57.1%)
**Regimen**	
Short Term Regimen	117 (20.7%)
Long Term Regimen	457 (53.4%)
Individualised regimen	162 (18.9%)
modified Short Term Regimen/ Standard regimen	60 (7.0%)
**TB Registration Group**	
New	454 (53.0%)
Retreatment	402 (47.0%)
**Place of Directly Observed Treatment**	
Initiation Facility based	112 (13.1%)
Follow Up Facility	744 (86.9%)
**Received Food Supplies**	
No	166 (19.4%)
Yes	690 (80.6%)
**Received Transport**	
No	89 (10.4%)
Yes	767 (89.6%)
**Taking alcohol**	
No	569 (66.5%)
Yes	287 (33.5%)
**Adherence to treatment**	
Missed dozes	291 (34%)
All dozes taken	565 (66%)
**Clinical Visits made**	
Missed	351 (41%)
All Attend	505 (59%)

### Prevalence of AEs among MDR-TB patients on second line anti TB treatment

Out of 856 MDR-TB patients, 369/856 (43.1%) had AEs out of these 145/856 (16.9%) suffered from more than one AE; 106/856 (12.4%) suffered from two AEs, 27/856 (3.2%) suffered from three AEs and 12/856 (1.4%) suffered from more than three AEs.

Most of the patients, 244/856 (29%) suffered from arthralgia; 204/244 (83.6%) were mild, 38/244 (15.6%) were moderate, and only 2/244 (0.8%) were severe. 75/856 (9%) of the patients had ototoxicity; 19/75 (25.3%) had mild ototoxicity; 18/75 (24%), moderate ototoxicity, 24/7/5 (32.2%), severe ototoxicity; and 14/75 (18.7%) had life-threatening ototoxicity. Patients with peripheral neuropathy (29/856, 3%), dermatologic disease (36/856, 4%), nausea and vomiting (58/856, 7%), psychiatric/psychosis (15/856, 2%), vision change (17/856, 2%), and gastrointestinal disease (37/856, 4%) had mild, moderate, and severe disease. Patients who suffered from gynecomastia (7/856, 3%) were mild and moderate. Patients who suffered from hypothyroidism and hepatotoxicity had only moderate effects, as seen in Table 2.

**Table 2:** A table showing percentages of adverse events for the MDR-TB patients on second line anti-TB drugs

Adverse Events	Mild	Moderate	Severe	Life threatening	Total
**Arthralgia**	204 (83.6%)	38 (15.57%)	2 (0.8%)		(29%) 244/856
**Peripheral Neuropathy**	22 (75.9%)	6 (20.7%)	1 (3.4%)		(3%) 29/856
**Gynecomastia**	5 (71.4%)	2 (28.6%)			(1%) 7/856
**Dermatologic**	12 (33.3%)	22 (61.1%)	2 (5.5%)		(4%) 36/856
**Ototoxicity**	19 (25.3%)	18 (24%)	24 (32%)	14 (18.7%)	(9%) 75/856
**Gastrointestinal**	14 (37.8%)	22 (59.4%)	1 (2.7%)		(4%) 37/856
**Hypothyroidism**		6 (100%)			(1%) 6/856
**Hepatotoxicity**		1 (100%)			(0.1%) 1/856
**Nausea / Vomiting**	17 (29.3%)	37 (63.8%)	4 (6.9%)		(7%) 58/856
**Psychiatric / psychosis**	5 (33.3%)	4 (26.7%)	6 (40%)		(2%) 15/856
**Vision change**	14 (82.3%)	2 (11.8%)	1 (5.8%)		(2%) 17/856

### To determine the factors associated with AEs of MDR-TB treatment among MDR-TB patients in Mulago national and Mbarara regional referral hospitals in Uganda

From Table 3 below; patients that received food supplies were 39% less likely to suffer from AEs compared to those that did not receive the food supplies at adjusted (Adj) PR 0.39; 95% CI (0.51–0.71). Patients that did not receive transport to attend their monthly clinical visits were 90% more likely to suffer from AEs compared to those receiving the transport Adj PR 1.9; 95% CI (1.36-3.00). 20% of the patients that took alcohol were more likely to suffer from AEs compared to those that did not consume alcohol at Adj PR 1.2; 95% CI (1.05–1.43).

Patients that received their treatment from follow up facilities were 60% more likely to suffer from AEs compared to those that received their daily treatment from the initiation facilities at Adj PR 1.6; 95% CI (1.10–2.41). Patients who received the 24 months’ regimen were 40% more likely to suffer from AEs compared to those that were on the short-term regimen at Adj PR 1.4; 95% CI (1.07–1.76) controlling for other factors. Patients that received an individualized regimen were 50% more likely to suffer from AEs compared to those that were on the short-term regimen at Adj PR 1.5; 95% CI (1.11–1.93) controlling for other factors.

**Table 3:** A table showing unadjusted and adjusted prevalence ratios for factors associated with adverse events for the MDR-TB patients on second line anti-TB drugs

Variables	No AEs	AEs	Unadjusted		Adjusted PR	
			PR	95% CI	PR	95% CI
**Number of Patients**	487 (56.9%)	369 (43.1%)				
**Age**						
0–14	29 (5.9%)	10 (2.7%)	1			
15–24	78 (16%)	55 (14.9%)	1.6	(0.91–2.85)		
25–34	162 (33.3%)	129 (34.9%)	1.7	(0.99–2.99)		
35–44	128 (26.3%)	102 (27.6%)	1.7	(0.99–3.01)		
45–54	61 (12.5%)	53 (14.3%)	1.8	(1.02–3.21)		
55+	29 (5.9%)	20 (5.4%)	1.5	(0.84–2.99)		
**Weight**						
1-29.9 kg	54 (11.1%)	23 (6.2%)	1			
30-35.9 kg	18 (3.7%)	9 (2.4%)	1.1	(0.59–2.11)		
36-45.9 kg	103 (21.1%)	86 (23.3%)	1.5	(1.04–2.21)		
46-55.9 kg	171 (35.1%)	149 (40.4%)	1.5	(1.08–2.23)		
56-69.9 kg	117 (24.0%)	84 (22.7%)	1.3	(0.95–2.04)		
70 kg+	24 (4.9%)	42 (4.9%)	1.4	(0.88–2.34)		
**Sex**						
Male	316 (64.9%)	227 (61.5%)	1			
Female	171 (35.1%)	142 (38.5%)	1.1	(0.92–1.27)		
**Marital Status**						
Single	201 (41.3%)	153 (41.5%)	1			
Married	199 (40.9%)	151 (41%)	0.9	(0.84–1.18)		
Separated	87 (17.9%)	65 (17.6%)	0.98	(0.79–1.23)		
**Occupation**						
Working	360 (73.9%)	273 (73.9%)	1			
Not working	127 (26.1%)	96 (26.1%)	0.9	(0.83–1.18)		
**Form of employment**						
Formal	22 (6.1%)	23 (8.4%)	1			
Informal	339 (93.1%)	250 (91.6%)	0.8	(0.61–1.12)		
**Received Food supplies**						
No	74 (15.2%)	92 (24.9%)	1		1	
Yes	413 (84.8%)	277 (75.1%)	0.7	(0.61–0.85)	0.61	(0.51–0.71) ***
**Received Transport**						
Yes	69 (14.2%)	20 (5.4%)	1		1	
No	418 (85.8%)	349 (94.6%)	2	(1.36-3.00)	1.9	(1.21–3.11) ***
**Drug Abuse (Alcohol)**						
No	350 (71.9%)	219 (59.4%)	1		1	
Yes	137 (28.1%)	150 (40.6%)	1.3	(1.16–1.58)	1.2	(1.05–1.43) **
**Co-Infected with HIV**						
No	211 (43.3%)	156 (42.3%)	1			
Yes	276 (56.7%)	213 (57.7%)	1.02	(1.07–1.19)		
**Regimen**						
STR	121 (24.8%)	56 (15.2%)	1		1	
LTR	236 (48.6%)	221 (59.9%)	1.5	(1.20–1.93)	1.4	(1.07–1.76) *
IND	89 (18.2%)	73 (19.8%)	1.4	(1.08–1.87)	1.5	(1.11–1.93) **
m STR/STD	41 (8.4%)	19 (5.1%)	1.001	(0.65–1.53)	1.1	(0.69–1.64)
**Adherence to treatment**						
Missed dozes	179 (36.8%)	112 (30.3%)	1			
All dozes taken	308 (62.2%)	257 (69.7%)	1.2	(0.99–1.40)		
**Clinical Visits made**						
Missed	192 (39.4%)	159 (43.1%)	1			
All Attend	295 (60.6%)	210 (56.9%)	0.9	(0.78–1.07)		
**Place of DOT**						
Initiation Site	85 (17.4%)	27 (7.3%)	1		1	
Follow Up Facility	402 (82.6%)	342 (92.7%)	1.9	(1.36–2.67)	1.6	(1.10–2.41) *
**TB Reg Group**						
New	255 (52.4%)	199 (53.9%)	1			
Retreatment	232 (47.6%)	170 (46.1%)	0.9	(0.82–1.12)		

* - p < 0.05 ** - p < 0.01 *** - p < 0.001

kg – kilogram, STD - standard treatment STR- short term regimen (9–12 months), LTR- long term regimen (20–24 months), IND- Individualised regimen, mSTR- modified short term regimen and DOT- directly observed therapy. Reg - registration

## Discussion

### Summary of the results

The study determined the prevalence of adverse events in two referral hospitals, where 43.1% of MDR-TB patients had AEs and 16.9% suffered from more than one AE. Furthermore, the study determined the factors associated with AEs, and these included patients started on the 24 months regimen (LTR) and individualized regimens being more likely to suffer from AEs. Lack of transport for clinical monitoring, alcohol consumption, and receipt of directly observed therapy from peripheral health facilities were significantly associated with experiencing AEs. However, patients who received food supplies were less likely to suffer from AEs.

### Prevalence of AEs

The purpose of the study was to determine the prevalence and factors associated with AEs of MDR-TB treatment in Mulago NRH and Mbarara RRH among MDR-TB patients. The above results showed that 43.1% of the MDR-TB patients suffered from AEs; and 16.9% suffered from more than 2 adverse event. Compared to the prevalence of AEs associated with MDR-TB treatment in India, at 57.6%, this is higher than that of 43.1% found in this study because of the high prevalence of MDR-TB patients in India. [17]. The prevalence is relatively high and if these AEs are not well managed, the affected patients may end up being lost to follow up (LFU) which may lead to extensively drug resistant TB (XDR-TB).

In this study, patients who were co-infected, particularly with HIV, experienced AEs from MDR-TB treatment in 57.1% of cases. This may be because of the high pill burden that the patient has and the drug interactions between the two diseases [18]. These results were lower compared to the findings of a systematic review that showed 83.7% of the HIV/MDR-TB patients suffering from AEs [19].

In this study, most patients had mild forms of AEs 83.6% and the most common AEs were joint pain (arthralgia) and hearing loss (ototoxicity). kanamycin, an injectable agent included in the majority of patients’ regimens, has been linked to ototoxicity [20]. Furthermore, 14 of the patients in the study had life-threatening forms of AE due to hearing loss. This was because Kanamycin normally affects the ears. These results are similar to a study [21] that showed patients on second line anti-TBs mostly suffered from joint pain (arthralgia). Ototoxicity was the most severe AE with 32% of the patients having it, these results are higher than a study that showed that 44% of them having ototoxicity and 14% had to change treatment because of the severity [22].

### Factors associated with AEs

In this study, age was not statistically significant for AEs which was contrary to the findings that showed age was significant especially for those that were 40 years above [23]. This is because it was a case-control study and had a higher sample size compared to this one. The age groups most affected were (25–34, 35–44) with a mean of 34 years and 12.4 SD with about 61.5% being males with AEs. The reason for this, is because these are the most economically active age groups that strive hard to make ends meet. Therefore, the chances of exposure are high since they interact with individuals that smoke, work in mines which are risk groups for TB. Uganda is named among the TB/HIV high burdened countries according to the World Health Organization (WHO) [9].

The provision of food to patients on second-line anti-TB treatment helps reduce the risk of AEs. This is because taking MDR-TB drugs after a meal or food reduces AEs such as nausea, vomiting and irritations in the stomach. Since most MDR-TB patients may not be able to afford a meal daily, it’s important to provide food to them to reduce the risk of AEs from the drugs. The study findings were similar to findings that showed providing food supplies was statistically significant to adherence to treatment and good treatment outcomes [24].

The findings in this study showed that patients taking alcohol were 20% more likely to suffer from AEs because the treatment caused them depression and a lot of pain. A study showed that 14% of the patients were likely to have depression and sleeping disturbance as AEs while on MDR-TB treatment which is similar to the findings in this study [25].

Patients who were receiving DOT from the follow up facilities were most likely to suffer from AEs. This is because health workers at follow up facilities may not be as well trained as those at the initiation facilities in managing the AEs. The untrained health workers at the follow-up facilities may mismanage the patients due to limited training which may lead to AEs. At the follow up facilities they may not have the facilities and equipment to diagnose the AEs and prevent them early enough [26].

The study findings further showed who patients that were taking long term regimens (18–24 months) and individualized regimens for MDR-TB were significantly associated with AEs. This is contrary to the study findings that showed MDR-TB treatment regimens weren’t associated to AEs [27]. Despite similar results in patients receiving kanamycin, which is used in a long-term regimen, they were 98% more likely to experience ototoxicity[21].

### Study Limitations

The study used secondary data from a clinical setting. Such data were not comprehensive to include some key variables that have been associated with AEs, for example socio-economic status. In addition, the study did not consider the levels of care for the different health facility since only referral hospitals were included. This potentially created selection bias with patients reporting to lower-level facilities being left out. This affects the generalizability of the findings. Further, the study did not assess AEs that were due to other drugs for example ARVs. Therefore, our findings of this study should be interpreted in this context.

## Conclusion

The overall prevalence of adverse events among MDR-TB patients is high at 43.1% with about 17% having multiple AEs. The provision of food supplies and transport to patients were associated with a reduced likelihood of reporting AEs. Patients taking alcohol while on treatment had high chances of reporting AEs. We recommend the provision of transportation and nutritious food to all MDR-TB patients to alleviate AEs and eventually promote adherence to treatment. Health promotion programs in healthcare facilities should also be emphasized.

## Data Availability

The dataset extracted and analyzed during the study are not publicly available due to restricted access of patient data from the MDR-TB database from ministry of health Uganda/ national TB and leprosy program but the data is available from the corresponding author on reasonable request.

## References

[1] Defeat-TBreport. USAID Defeat TB Annual Report Oct 1, 2018 - Sept 30., 2019. 2019.

[2] Kendall EA, Theron D, Franke MF, Van Helden P, Victor TC, Murray MB (2013). Alcohol, hospital discharge, and socioeconomic risk factors for default from multidrug resistant tuberculosis treatment in rural South Africa: a retrospective cohort study. PLoS ONE.

[3] WHOreport. The WHO global task force on TB impact measurement. 2019.

[4] National. -TB-and-Leprosy-Program-Ministry-of-Health-Uganda. Uganda National Guidelines for the programmatic management of Drug-Resistant Tuberculosis. 2016;Second Edition.

[5] Furin JJ, Mitnick CD, Shin SS, Bayona J, Becerra MC, Singler JM (2001). Occurrence of serious adverse effects in patients receiving community-based therapy for multidrug-resistant tuberculosis. Int J tuberculosis lung disease: official J Int Union against Tuberculosis Lung Disease.

[6] Buziashvili M, Mirtskhulava V, Kipiani M, Blumberg HM, Baliashvili D, Magee MJ (2019). Rates and risk factors for nephrotoxicity and ototoxicity among tuberculosis patients in Tbilisi, Georgia. Int J tuberculosis lung disease: official J Int Union against Tuberculosis Lung Disease.

[7] Shin SS, Pasechnikov AD, Gelmanova IY, Peremitin GG, Strelis AK, Mishustin S (2007). Adverse reactions among patients being treated for MDR-TB in Tomsk, Russia. Int J tuberculosis lung disease: official J Int Union against Tuberculosis Lung Disease.

[8] Meressa D, Hurtado RM, Andrews JR, Diro E, Abato K, Daniel T (2015). Achieving high treatment success for multidrug-resistant TB in Africa: initiation and scale-up of MDR TB care in Ethiopia—an observational cohort study. Thorax.

[9] WHOreport. WHO releases new global lists of high-burden countries for TB, HIV-associated TB and drug-resistant TB. 2020.

[10] USAIDreport. UGANDA TUBERCULOSIS ROADMAP OVERVIEW, FISCAL YEAR 2021. 2021.

[11] WHOguidelines. Companion handbook to the WHO guidelines for the programmatic management of drug-resistant tuberculosis. World Health Organization; 2014. 25320836

[12] WHOreport. MDR-TB Short term regimen. 2016.

[13] Kalandarova L, Tillashaikhov M, Parpieva N, Saidova S, Gadoev J, Alikhanova N (2016). Treatment outcomes and adverse reactions in patients with multidrug-resistant tuberculosis managed by ambulatory or hospitalized care from 2010–2011 in Tashkent, Uzbekistan. Public health panorama.

[14] Mody K, Kubal M. Spectrum of adverse drug reactions during MDR TB treatment. Eur Respiratory Soc; 2013.

[15] Arnold A, Cooke GS, Kon OM, Dedicoat M, Lipman M, Loyse A (2017). Adverse effects and choice between the injectable agents amikacin and capreomycin in multidrug-resistant tuberculosis. Antimicrob Agents Chemother.

[16] Prasad R, Singh A, Gupta N (2019). Adverse drug reactions in tuberculosis and management. indian J tuberculosis.

[17] Dela AI, Tank ND, Singh AP, Piparva KG (2017). Adverse drug reactions and treatment outcome analysis of DOTS-plus therapy of MDR-TB patients at district tuberculosis centre: a four year retrospective study. Lung India: Official Organ of Indian Chest Society.

[18] Mukonzo J, Aklillu E, Marconi V, Schinazi RF (2019). Potential drug–drug interactions between antiretroviral therapy and treatment regimens for multi-drug resistant tuberculosis: implications for HIV care of MDR-TB co-infected individuals. Int J Infect Dis.

[19] Schnippel K, Firnhaber C, Berhanu R, Page-Shipp L, Sinanovic E (2017). Adverse drug reactions during drug-resistant TB treatment in high HIV prevalence settings: a systematic review and meta-analysis. J Antimicrob Chemother.

[20] Poka-Mayap V, Pefura-Yone E, Kuaban C (2020). Kanamycin-induced ototoxicity during treatment of multidrug-resistant tuberculosis. Rev Mal Respir.

[21] Modongo C, Sobota RS, Kesenogile B, Ncube R, Sirugo G, Williams SM (2014). Successful MDR-TB treatment regimens including amikacin are associated with high rates of hearing loss. BMC Infect Dis.

[22] Van der Walt M, Lancaster J, Odendaal R, Davis JG, Shean K, Farley J (2013). Serious treatment related adverse drug reactions amongst anti-retroviral naïve MDR-TB patients. PLoS ONE.

[23] Kocfa Revilla-Montag A, Guillen-Bravo S, Velez-Segovia E, Soria-Montoya A, Nuñez-Garbin A et al. Factors associated with anti-tuberculosis medication adverse effects: a case-control study in Lima, Peru.PloS one. 2011;6(11). 10.1371/journal.pone.0027610 PMC3217998 22110689

[24] Ciobanu A, Domente L, Soltan V, Bivol S, Severin L, Plesca V (2014). Do incentives improve tuberculosis treatment outcomes in the Republic of Moldova?. Public Health Action.

[25] Bezu H, Seifu D, Yimer G, Mebrhatu T. Prevalence and risk factors of adverse drug reactions associated multidrug resistant tuberculosis treatments in selected treatment centers in Addis Ababa Ethiopia. Journal of Tuberculosis Research. 2014;2014.

[26] Kasozi S, Kirirabwa NS, Kimuli D, Luwaga H, Kizito E, Turyahabwe S (2021). Addressing the drug-resistant tuberculosis challenge through implementing a mixed model of care in Uganda. PLoS ONE.

[27] Gualano G, Mencarini P, Musso M, Mosti S, Santangelo L, Murachelli S (2019). Putting in harm to cure: drug related adverse events do not affect outcome of patients receiving treatment for multidrug-resistant tuberculosis. Experience from a tertiary hospital in Italy. PLoS ONE.

